# Quality of life in children suffering from headaches: a systematic literature review

**DOI:** 10.1186/s10194-023-01595-0

**Published:** 2023-09-18

**Authors:** S. Ombashi, E. Tsangaris, A. G. Heeres, V. van Roey, R. F. Neuteboom, M. L. C. van Veelen-Vincent, K. Jansson, I. M. J. Mathijssen, A. F. Klassen, S. L. Versnel

**Affiliations:** 1 Department of Plastic and Reconstructive Surgery, Erasmus MC, University Medical Center Rotterdam, Rotterdam, The Netherlands; 2 European Reference Network for Craniofacial Anomalies and Ear- Nose- and Throat Disorders, Erasmus University Medical Center, Dr. Molewaterplein 40, 3015 GD Rotterdam, The Netherlands; 3 Brigham and Women’s Hospital, Harvard Medical School, Boston, MA USA; 4 Children’s Brain Lab, Erasmus MC, University Medical Center Rotterdam, Rotterdam, The Netherlands; 5 Department of Neurology, Erasmus MC, University Medical Center Rotterdam, Rotterdam, The Netherlands; 6 Department of Pediatric Neurosurgery, Erasmus MC, University Medical Center Rotterdam, Rotterdam, The Netherlands; 7 Department of Reconstructive Surgery and Craniofacial Surgery, Stockholm, Karolinska University Hospital, Stockholm, Sweden; 8 Department of Pediatrics, McMaster University, Hamilton, ON Canada

## Abstract

**Background:**

Headaches are the most common complaints among pediatric populations. Determining the cause and appropriate treatment for headaches may be challenging and costly, and the impact of headaches on the lives of patients and their families is not well understood.

**Objective:**

A systematic literature review was conducted to examine what PROMs are currently used, and to identify quality of life (QoL) concepts important to children suffering from headaches and any known determinants of QoL.

**Methods:**

Embase, Medline, Web of Science, CINAHL, EBSCOhost, PsychINFO, Cochrane CENTRAL and Google Scholar were searched from their inception through to June 2021. Studies investigating QoL, using a validated outcome measure in pediatric patients with headaches, were included. Relevant studies were identified through title and abstract screening and full text review by two independent reviewers. A citation review of included studies was performed. QoL concepts were extracted from the outcome measures that were used in each study to develop a preliminary conceptual model of QoL in children suffering from headaches. Determinants of QoL were also identified and categorized.

**Results:**

A total of 5421 studies were identified in the search. Title and abstract screening resulted in the exclusion of 5006 studies. Among the 415 studies included for full text review, 56 were eligible for final analysis. A citation review resulted in the addition of five studies. Most studies were conducted in high-income countries and included a patient-sample accordingly (*n* = 45 studies). Sixteen different PROMs were identified in the included studies, of which the PedsQL was used the most often (*n* = 38 studies).

The most common health concepts reported were physical functioning (*n* = 113 items), social and psychological wellbeing (*N* = 117, *n* = 91 resp.). Twenty-five unique determinants of QoL were extracted from the included studies.

**Conclusion:**

There is a need for a condition-specific PROM to facilitate the measurement of QoL outcomes in the pediatric headache population. A conceptual model was developed based on the findings from the health concepts. Findings from this review could be used for future qualitative interviews with pediatric patients with headaches to elicit and refine important QoL concepts.

**Supplementary Information:**

The online version contains supplementary material available at 10.1186/s10194-023-01595-0.

## Introduction

Headaches impose a significant negative impact on the quality of life (QoL) of individuals of all ages [1]. The global burden of headaches on QoL was emphasized in a 2019 report by the Global Burden of Disease [2]. Among children (aged ≤ 18 years), headaches were the most common complaint, reported in 56–58% of the population globally [1, 3]. Through puberty and adolescence, prevalence increase to 65%, with over 90% of the patients with headaches reported a lifetime history of headaches [4].

Lifestyle interventions, including physical activity and weight management are recommended for symptom management and to prevent headaches [5, 6]. Preventive or acute pharmacological therapy may also be indicated when lifestyle changes fail to alleviate symptoms – although identifying the balance between adequate dosage and possible side-effects can be difficult [5, 7, 8]. Surgical interventions are rarely indicated, but are used in cases where vascular anomalies or craniosynostosis are present [9, 10].

Few studies investigate the impact of headaches on the QoL of children. Those that do, report an important impact on their QoL depending on the severity and frequency of symptoms, including their physical functioning, school performance, psychological well-being, and participation in social activities [11, 12]. Presently, outcomes of headaches and their treatment in children are measured using objective clinical evaluations, including neuroimaging, laboratory analyses, headache frequency, and days missed from school [13, 14]. Given the impact that headaches can have on the QoL of children, there is a need to evaluate outcomes from the patient perspective. To systematically assess QoL in children with headaches, a valid, reliable, and condition-specific patient reported outcome measure (PROM) is needed. The purpose of this systematic literature review was to identify PROMs currently used in children diagnosed with headaches. A secondary aim was to extract QoL concepts measured by the PROMs, and known determinants of QoL from the included studies.

## Methods

### Search strategy

The Preferred Reporting Items for Systematic Reviews and Meta-Analyses (PRISMA) statement was used to guide the reporting of this systematic literature review [15]. Embase, Medline, Web of Science, CINAHL, EBSCOhost, PsychINFO, Cochrane Central, and Google Scholar were searched from their inception through to November 2022. Search terms were determined by members of the clinical and research team and were presented to a medical librarian who developed the search strategy for each database. Detailed search strategies for each database are provided in Appendix 1.

### Screening process

Studies that reported using a validated PROM to evaluate QoL outcomes of pediatric patients with headaches, aged ≤ 18 years, in any language, were included. Studies that collected data from pediatric and adult patients simultaneously, or from patients with other conditions, were included if the results of the pediatric patients with headaches were reported separately. Conference reports, abstract and reviews were excluded. Studies were also excluded if they used a PROM with no published evidence of their development, validity, and reliability.

Search results for each database were merged within Endnote. Title, abstract, and full-text screening was performed by two independent reviewers according to the blinded method of Bramer et al. [16]. A third reviewer was assigned to resolve any disagreement. A citation review of included studies and reviews was performed to identify any additional studies for inclusion. When necessary, the corresponding author for candidate studies was contacted to clarify eligibility or to obtain missing information.

### Data collection

Full-text of included studies were reviewed in detail to extract the following information: first author last name, year of publication, type of headaches evaluated, diagnostic criteria, patient characteristics (gender, age), sample size, comparison sample (if applicable), name and type of outcome measure used, and whether or not the study excluded patients with psychiatric comorbidities. Furthermore, the country of origin of the patient-sample was registered. Countries were classified according to the World Bank Classification [17]. One reviewer extracted the details of the study and a second reviewer checked the data extraction.

### Concept sort

The original copies of the PROMs that were used to evaluate QoL outcomes in pediatric patients with headaches were obtained, together with the publications describing their development and validation. A concept sort was conducted to identify important health concepts to pediatric patients with headaches: All items of each PROM were extracted and collected into a Microsoft Excel worksheet for coding. Each item was then labelled with a top-level code and one or more subcodes. Coding was done independently by two research team members (SO, ET). To review coding, and to resolve possible discrepancies, the research team planned several meetings. PROMs that could not be retrieved were excluded from the concept sort. Items from each PROM were extracted and entered into a Microsoft Excel worksheet. Items were coded by applying a top-level code and one or more sub-codes. Coding was conducted by two researchers, who worked independently. Disagreements were resolved through discussion. The methodological quality of the pediatric headache-specific PROMs was assessed using the Consensus-based Standards for the selection of health Measurement Instruments (COSMIN) risk of bias checklist [18].

### Determinants

Identifying determinants is important for further understanding of variability in QoL outcomes in pediatric headache populations. Demographic, environmental and socioeconomic variables such as gender, age, and family income, together with individual variables form a complex interaction with the child’s QoL and are worthy of study [19, 20]. In order to consider factors of the child as an individual, family characteristics and the larger community environment, determinants that described a relation with one or more QoL domain(s) were extracted from the included studies. The p-value for the relationship with the QoL domain was also extracted, and was stated as significant where the value was less than 0.05. Information was checked by a second reviewer and determinants were sorted into categories.

## Results

**Fig. 1:**
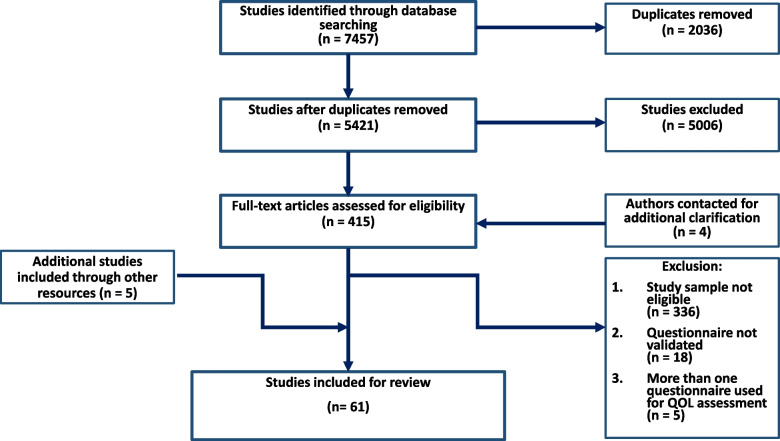
PRISMA flowchart of the studies included

A total of 5421 studies were retrieved from our search (Fig. 1).

After title and abstract screening, 415 studies were eligible for full text review. After full text review, 359 studies were excluded, mostly as their study sample did not fit the criteria (*n* = 336 studies, Fig. 1). Citation review of included studies and reviews identified five additional studies that were eligible for inclusion (Fig. 1). For an overview, Table 1 summarized the characteristics from the included studies, whilst Table 2 outlines all individual study characteristics. The age of the children in each study ranged from two to eighteen years and sample sizes ranged between seven to 10,677 patients. A total of 31 studies compared outcomes of pediatric patients with headaches with a normative sample (Table 1). One study included an adult sample besides their pediatric sample, and analyzed them separately (Table 2).

**Table 1:** Summarizing characteristics

**Total number of studies**	61 studies
**Year of publication (range)**	1999 – 2022
**Sample size (range)**	7 – 10,677 patients
**Comparison sample available (n of studies)**	31 studies
Exclusion of patients with psychiatric comorbidities **(n of studies)**	17 studies
**Age of patients included (range)**	2 – 18 years of age
**Including patients from high-income countries (n of studies)**	45 studies
**Including patients from upper-middle-income countries (n of studies)**	13 studies
**Including patients from lower-middle-income countries (n of studies)**	2 studies

**Table 2:** Characteristics of included studies

*Author*	Year	Country	Type of headache	Diagnosic criteria	Gender (%) Female-male	Age range (in years)	Sample size	Comparison sample	QoL questionnaire used	Patient-, parent or both reported	Exclusion psychiatric comorbidities
Al-Hashel [21]	2020	Kuwait	PH	ICHD-III crit	69–31	7 to 17	466	N/A	HARDSHIP	Patient	No
Açıkel [22]	2021	Turkey	TTH, M	ICHD-II	42–58	8 to 18	59	Normative sample	HARDSHIP	Patient	Yes
Amouroux [23]	2017	France	M, TTH	ICHD-II crit	43–58	4 to 18	73	N/A	VSP-A	Patient	No
Bai [24]	2017	Netherlands	M, SH	Parent reported	57–43	4 to 11	77	Normative sample, asthma, eczema, ADHD, dyslexia	CHQ-PF28	Parent	Yes
Benore [25]	2018	USA	CM, cTTH, daily headache	ICHD-II crit	74–26	up to 18	135	N/A	PedsQL	Both	No
Bruijn [26]	2009	Netherlands	M, cTTH	IHS class	NR	4 to 17	70	Normative sample, ADHD, asthma	CHQ-PF50	Parent	No
Caruso [27]	2022	USA	M, UdH	ICHD-III, or clinician-reported	76–24	10 to 17	88	N/A	PedsQL	Patient	No
Castro [12]	2013	Brazil	M, cDH, cTTH, eTTH	Episode > 3 months	61–40	7 to 14	185	Normative sample	PedsQL	Patient	No
Chen [28]	2007	Japan	NR	Episode > 6 months	NR	5 to 18	85	Normative sample	PedsQL	Both	Yes
Connelly [29]	2006	USA	M, CDH, eTTH	Neurologist	NR	7 to 12	40	Normative sample	PedsQL	Both	Yes
Cordova [30]b	2021	Germany	RH	Self-reported	37–63	11 to 17	2238	Normative sample	KINDL	Both	No
Gozubatik [31]	2021	Turkey	TTH, M	ICHD-III crit	39–61	8 to 18	61	Normative sample	PedsQL	Patient	Yes
De Tommaso [32]	2017	Italy	M	ICHD-III crit	56–44	8 to 15	151	N/A	PedsQL	Both	No
Dudeney [33]	2019	USA	cH	24 > episodes < 3 months	71–29	10 to 18	56	N/A	PedsQL-SF	Patient	No
Eren [34]	2021	Turkey	M	IHS class	65–35	8 to 18	43	Normative sample	PedsQL	Parent	Yes
Ferracini [35]	2013	Brazil	M	IHS class	NR	6 to 12	50	Normative sample	PedsQL	Both	No
Genc [36]	2021	Lithuania	M, TTH, pMOH, headache ≥ 15 days	Self-reported, ICHD-III crit	-	7 to 17	1858	Normative sample	HARDSHIP	Patient	No
Genizi [37]	2019	Israel	M	ICHD-III crit	59–41	8 to 12	54	Normative sample	PedsQL	Patient	No
Gerber [38]	2010	Germany	M, TTH	IHS class	65–35	7 to 16	34	N/A	KINDL	Both	No
Hainsworth [39]	2014	USA	M, CDH	Episode > 3 months	43–57	11 to 16	7	N/A	PedsQL	Both	No
Hao [40]	2010	China	M	Clinician-reported	50–50	5 to 18	618	Normative sample, leukemia, epilepsy, Gilles de la Tourette's syndrome	PedsQL	Both	No
Hesse [41]	2015	USA	RH	≥ 4 episodes/month for ≥ 3 months	100 (F)	11 to 16	15	N/A	PedsQL	Both	Yes
Kashikar-Zuck [42]	2013	USA	cM	ICHD-II crit	82–18	10 to 18	153	Juvenile fibromyalgia	PedsQL	Both	No
Kernick [43]	2009	UK	NR	Self-reported	NR	12 to 15	648	Normative sample	PedsQL, PedMIDAS	Patient	No
Koenig [44]	2013	Germany	M, CM, eTTH, cTTH	ICHD-II crit	78–22	12 to 17	71	N/A	KIDSCREEN-27	Patient	Yes
Kovacevic [45]	2017	Serbia	M	ICHD-III crit	53–47	7 to 17	32	N/A	KIDSCREEN-27	Both	Yes
Koller [46]	2019	Austria	M	ICHD-III crit	49–51	8 to 17	37	Normative sample	PedsQL	Patient	No
Krause [47]	2016	Germany	NR	Self-reported	NR	3 to 17	10.667	Normative sample	KIDSCREEN-10	Patient	No
Ladner [48]	2016	USA	NR	NR	NR	5 to 18	67	Normative sample	CHIP, HUI3	Both	No
Langeveld [49]	1999	The Netherlands	M	Clinician-reported	60–40	12 to 18	64	Normative sample	QLH-Y	Patient	No
Long [50]	2007	USA	M, TTH or Combined	NR	NR	8 to 10	44	Sickle cell disease, juvenile idiopathic arthritis	CHQ-PF50	Parent	No
Massey [51]	2008	Netherlands	NR	Self-reported	47–53	12 to 18	685	Normative sample	PedsQL-SF	Patient	No
McDonald [8]	2011	USA	M	ICHD-II crit	59–41	12 to 17	531	N/A	MSQ-A	Patient	No
Milde-Busch [52]	2010	Germany	M, TTH	IHS class	61–39	13 to 17	475	Normative sample	KINDL	Patient	No
Nodari [53]	2002	Italy	M, TTH	NR	55–45	10 to 18	310	Normative sample	QLH-Y	Patient	No
Orr [54]	2017	Canada	M	ICHD-II crit	68–32	10 to 18	85	Normative sample	PedsQL	Patient	Yes
Öztop [55]	2016	Turkey	M	IHS class	74–26	9 to 16	35	Normative sample	PedsQL	Both	No
Palermo [56]	2005	USA	M, TTH	NR	NR	13 to 16	45	Sickle cell disease, juvenile idiopathic arthritis	CHQ-CF87	Patient	No
Paniccia [57]	2020	Canada	UdH, M	NR	NR	8 to 12	231	Normative sample	KIDSCREEN-10 Index	NR	No
Petersen [58]	2009	Sweden	NR	Self-reported	NR	8 to 14	769	Normative sample	PedsQL	Both	No
Philipp [59]	2019	Austria	M, TTH, pMOH, UdH, headache ≥ 15 days/month	ICHD-III crit	60–40	10 to 18	2563	Normative sample	KIDSCREEN-10, -52 and -27	NR	No
Powers [60]	2003	USA	M, CDH	IHS class	55–45	2 to 18	572	Normative sample, cancer, rheumatologic disease	PedsQL	Both	No
Powers [61]	2004	USA	M	IHS class	57–43	2 to 18	686	Normative sample	PedsQL	Both	No
Rapoff [62]	2014	USA	M	ICHD-II crit	71–29	7 to 12	35	N/A	PedsQL	Both	Yes
Rettig [63]	2021	USA	CM	Treatment was indicated	21–79	10 to 17	135	N/A	PedsQL	NR	No
Riatha [64]	2013	Indonesia	PH	ICHD-II crit	60–40	13 to 17	75	Normative sample	PedsQL	Patient	No
Rocha-Filho [65]	2014	Brazil	PM, M, TTH, UdH	Clinician-reported	NR	10 to 15	NR	N/A	PedsQL	Patient	No
Sciruicchio [66]	2019	Italy	M, cM	ICHD-II crit	60–41	8 to 17	190	N/A	PedsQL	Both	Yes
Seeger [67]	2014	Canada	PTH	NR	67–33	13 to 17	15	N/A	PedsQL	Patient	No
Şentürk [68]	2022	Turkey	M, TTH, or both	ICHD-III crit	69–31	8 to 18	80	N/A	PedsQL	NR	No
Shaygan [69]	2021	Iran	cH	ICD-II crit	NR	12 to 18	NR	Musculoskeletal pain, other chronic pain	PedsQL	Patient	Yes
Shiri [70]	2013	Israel	M, cTTH	ICHD-II crit	30–70	10 to 18	10	N/A	PedsQL	Patient	Yes
Slater [71]	2012	USA	cDH	ICHD-II crit	78–22	10 to 17	169	N/A	PedsQL	Both	No
Talarska [72]	2017	Poland	M, CH, TTH	IHD-2004	59–41	8 to 18	173	Normative sample, epilepsy	PedsQL	Both	Yes
Talarska [73]	2007	Poland	M, TTH	NR	NR	8 to 18	64	N/A	PedsQL	Both	No
Todorov [74]	2009	USA	M	IHS class	75–25	11 to 17	63	Normative sample	PedsQL, PedMIDAS, MSQ-A	Both	Yes
Uneri [75]	2009	Turkey	M	ICHD-II crit	80–20	13 to 18	30	Normative sample	PedsQL	Both	Yes
Ung [76]	2018	USA	NR	NR	NR	7 to 17	84	N/A	PedsQL	Both	No
Witt [77]	2009	Germany	NR	NR	55–45	7 to 16	67	N/A	KINDL	Patient	No
Wober-Bingol [78]	2014	Austria, Turkey	M, MOH, TTH	ICHD-II crit	52–48	6 to 17	1.073	N/A	HARDSHIP	Patient	No
Yaghini [79]	2022	Iran	M	IHS class	36–64	5 to 15	72	N/A	PedMIDAS	Patient	No

*PH* Primary Headache, *(c)DH* (chronic) Daily Headache, *cH* Chronic Headache, *(p)MOH *probable Medication Overuse Headache, *(c/e)M* (chronic/episodic) Migraine, *(c/e) TTH* (chronic/episodic) Tension Type Headache, *RH *Recurrent Headaches, *UdH * undifferentiated headache, *NR* Not Reported, *N/A* Not Applicable

**Fig. 2:**
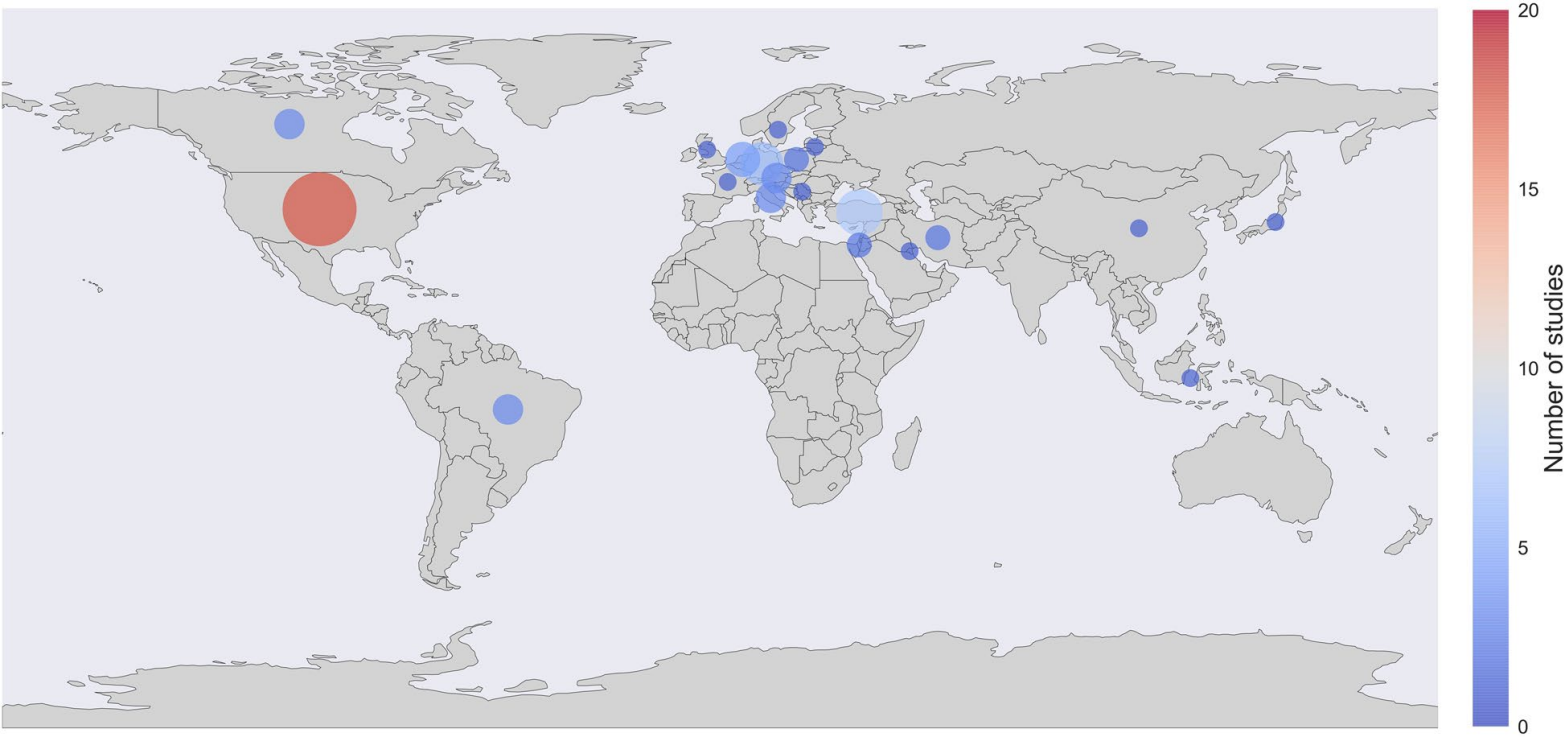
Choropleth presenting where patient samples from each included study derived from (note: authors of the study may derive from elsewhere)

Figure 2 outlines where each patient sample derived from.

The majority of studies (*n* = 45) included exclusively patient samples from high-income countries, such as the USA (*n* = 18 studies) and Western-Europe (*n* = 18 studies), as classified by the World Bank Classification [17] (Table 1, Fig. 2).

One study presented a sample from both high and an upper-middle income countries (Austria and Turkey [78]). Twelve studies were conducted in upper-middle income countries, namely Turkey [22, 31, 34, 55, 68, 75], Brazil [12, 35, 65], China [40], Serbia [45] and Indonesia [64]. Two studies examined patients from Iran, currently classified as a lower-middle income country [69, 79]. There were no studies that included patients from low-income countries (Table 2).

Headaches were mostly diagnosed based on international standardized classifications (Table 2). Most studies included patients suffering from migraine (*n* = 45 studies) or tension type headaches (*n* = 22 studies). Seventeen studies explicitly excluded patients suffering psychiatric (co)morbidities (Table 1).

**Fig. 3:**
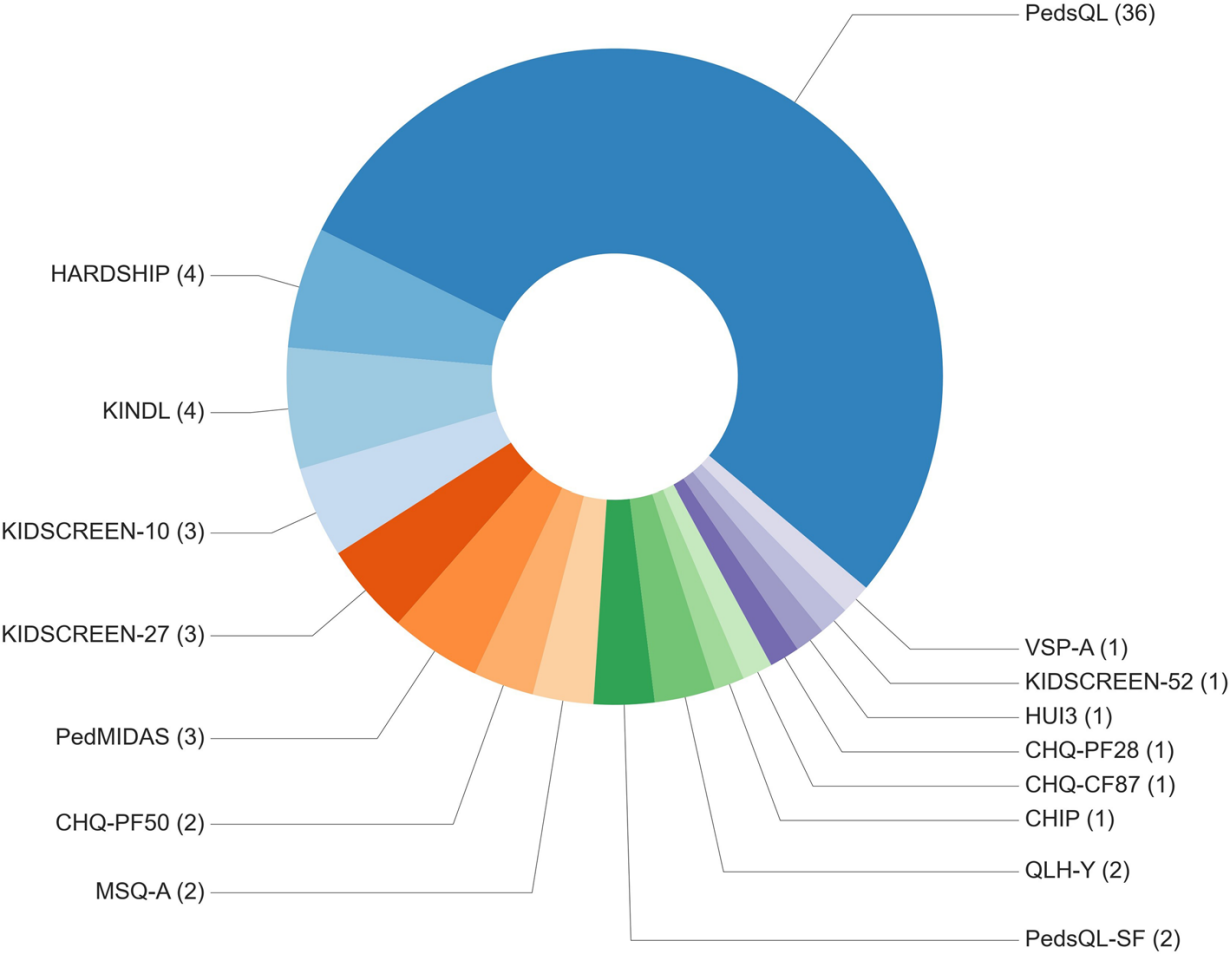
Overview of all PROM questionnaires used in the included studies, and the number of times that they were used. HARDSHIP: Headache-Attributed Restriction, Disability, Social Handicap and Impaired Participation. KINDL (German): Fragebogen für KINDer und Jugendliche zur Erfassung der gesundheitsbezogenen Lebensqualität (English translation: Questionnaire to assess Health-Related Quality of Life in children and adolescents). KIDSCREEN-52, -27, -10: health-related quality of life of children and adolescents aged 8 to 18 years (no acronym). PedMIDAS: Pediatric Migraine Disability Assessment Scale. CHQ-PF50, -PF 28; CHQ-CF28/CF87: Child Health Questionnaire. Either the Parent Form (PF), or the Child Form (CF). The numbers 28, 50 and 87 stand for the number of items each version contains. MSQ-A: Migraine-Specific Quality-of-Life Questionnaire, Adolescents. PedsQL (SF): Pediatric Quality of Life (Short Form). VSP-A (French): Vécu et Santé Perçue de l'Adolescent (English -free- translation: Perceived live and health, adolescent version). HUI3: Health Utility Index (mark three). CHIP: Coping Health Inventory for Parents. QLH-Y: Quality of Life Headache in Youth

An overview of all QoL PROMS identified is shown in Fig. 3.

Sixteen PROMs were identified. Most studies included the PedsQL, that was used in 38 studies to measure QoL in their patient sample, of which the Short form (SF) was used twice (Fig. 3). All other PROMs were used infrequently, i.e., five PROMs were used in three or four studies, and ten of the sixteen PROMs were used in one or two studies (Fig. 3). In four studies, more than one type of questionnaire was used for QoL assessment (Table 2).

### Concept sort for important health concepts

**Fig. 4:**
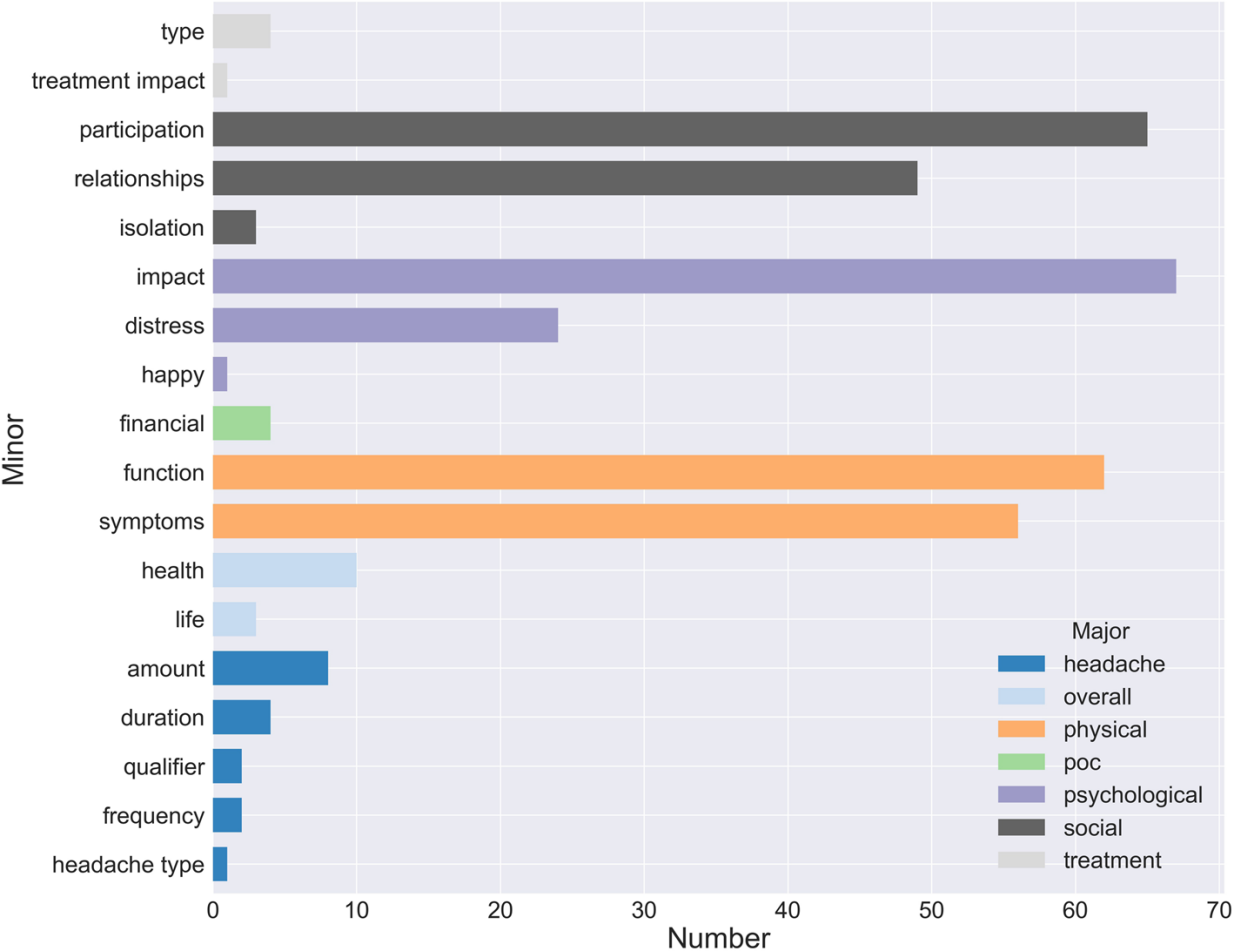
Concept sort of all items identified though the included questionnaires. Y-axis: the sublevel codes, or minor concepts. In the legend, top-level codes (of concepts) are summed up. X-axis: the number of items that were labeled with the minor concept

The most common health concepts reported were social and psychological wellbeing, and physical functioning. Findings of these concepts are reported in this section. Figure 4 includes all health concepts identified.

### Social function

Social function was the most-examined concept in the included studies. This concept was measured by 117 different items from ten PROMs. Participation (in society) was the most common sub-code (*n* = 65 items) [e.g. “During the past four weeks, to what extent has your physical health or emotional problems interfered with our normal social activities with family, friends, neighbors, or groups?”]. Sub-concepts asked about participation in leisure activities (*n* = 24 items); school (*n* = 23 items); work (*n* = 8 items), peers (*n* = 4 items) and hobbies (*n* = 4 items).

Furthermore, 49 items were sub-coded as relations. They mostly assessed emotional support (*n* = 19 items), and the social impact (*n* = 28 items) patients experienced. Items involved family (*n* = 18 items), friends (*n* = 11 items) and peers (*n* = 7 items).

### Physical health

Physical health was measured by 113 items in ten PROMs. Function was the most common sub-code within this concept, measured by 62 items. Twenty-four items were further coded as activities of daily living [e.g., “I need help dressing or bathing”, “How satisfied are you with your ability to perform your daily living activities?”]. Physical symptoms were measured by 54 items, of which 17 items were related to pain [e.g., “Last week I was troubled by eye pain when reading”, “I have pain in my neck/shoulders”].

### Psychological health

Ten different PROMs included 91 items that assessed the concept of psychological health. Psychological impact was the most common emerging sub-code, found in 67 items [e.g. “Thinking about the last week has your life been enjoyable?”]. Psychological distress was another common sub-code, measured in 23 items. All items within the latter sub-concept were related to anxiety. An example item coded under psychological distress is “During the past week I felt scared or unsure of myself”.

### Risk of bias assessment

Twelve generic QoL PROMs were used 55 times in 53 studies (Fig. 3, Table 1). Three QoL PROMs were identified through the studies that were targeted at pediatric patients with headaches, namely the Migraine specific quality of life questionnaire – Adolescent (MSQ-A); the Headache-Attributed Restriction, Disability, Social Handicap and Impaired Participation questionnaire (HARDSHIP); and the Quality of Life Headache in Youth (QLH-Y) (Fig. 3). The questionnaires were used solely or in combination with a generic PROM in a total of eight studies [8, 21, 22, 36, 49, 53, 74, 78].

A fourth questionnaire targeted at pediatric patients with headaches was identified, namely Pediatric Migraine Disability Assessment (PedMIDAS) (Fig. 3). Three studies used PedMIDAS as a tool to measure QoL, either combined with other QoL PROMs [43, 74], or as the only PROM [79]. However, PedMIDAS was not developed nor validated as a questionnaire measuring QoL, but is a (valid) six-item questionnaire to assess disability in childhood and adolescent headaches [80]. Therefore, PedMIDAS was not assessed with the COSMIN risk of bias checklist in this review.

To examine validity and reliability, the COSMIN risk of bias checklist was used to assess the MSQ-A, HARDSHIP and QLH-Y [18]. Information regarding the development processes of the PROMs was retrieved. However, not all information needed for complete assessment with the COSMIN risk of bias checklist was available. Consequently, no total score could be calculated, and the tool was used as a guideline rather.

The **MSQ-A** was developed from focus groups with adolescent patients with headaches. It did not include patients < 11 years [81]. The items were not developed for a pediatric population, but items were derived from the MSQ 2.1, an adult questionnaire, meaning the MSQ-A lacks the involvement of a target population for which the PROM was validated up to the point that items have already been generated [81].

The lack of a target sample was also found in the **HARDSHIP** questionnaire, of which the items derive from the adult version of the questionnaire as well [78]. In addition, items from general questionnaires such as the (PedMIDAS, KINDL) were included to this questionnaire [78]. Furthermore, it was found that the questionnaire primarily assesses headache impact rather than QoL [78]. Since it has included 12 items regarding QoL, the assessment of QoL is quite compact.

The questionnaire with the most extensive development process was the **QLH-Y**. Items were reviewed by a panel of eight youngsters and factor analyses/homogeneity analyses were performed [82]. However, only five patients with headaches were interviewed for the development of the questionnaire, and results were solely used for one domain (‘functional status’) [82]. Although the questionnaire was evaluated with a headache sample and a control group after validation, factor analyses/homogeneity analyses were performed on a normative adolescent sample instead of the entire target population [82].

### Important determinants of QoL

Thirty-six articles examined one or more determinant(s) of QoL. A total of 25 unique determinants were identified and are detailed in Table 3. A significant relation between a determinant and QoL was found 54 times (Table 3).

**Table 3:** Determinants of QOL

Determinant	Nr. Of analyses conducted	Nr. Of times relationship appeared significant
**Patient characteristics**		
Gender [22, 35, 51, 53, 73, 74]	**6**	**3** [35, 51, 73]
Age [22, 51, 53, 61, 73, 74]	**6**	**3** [53, 61, 73]
**Psychological traits**		
Depressive symptoms [22, 42, 76]	**3**	**3** [22, 42, 76]
Negative affectivity [49]	**1**	**1** [49]
Negative life events [51]	**1**	**1** [51]
Stress [49]	**1**	**1** [49]
Anxiety [42, 65, 76]	**3**	**2** [42, 76]
Anger [76]	**1**	**1** [76]
Health-goal importance [51]	**1**	**0**
Goal-frustration [51]	**1**	**1** [51]
Cognitive coping strategies [51, 74, 76]	**3**	**2** [51, 76]
Health mindset [27]	**1**	**1** [27]
**Headache variables**		
Type of headache [26, 31, 36, 52, 53, 59, 65, 70, 73, 78]	**10**	**5** [36, 52, 59, 65, 78]
Headache frequency, activity [31, 43, 49, 51, 53, 61]	**6**	**2** [49, 51]
Severity, intensity [22, 29, 51, 53, 61, 65, 78]	**7**	**5** [22, 29, 53, 65, 78]
Duration [55, 61, 73]	**3**	**1** [73]
**Comorbidity and functional impairment**		
Disability [23, 29, 35, 37, 54, 55]	**6**	**5** [23, 29, 37, 54, 55]
Comorbidities [55, 58, 71]	**3**	**2** [58, 71]
Pain measurements [31, 55, 66]	**3**	**3** [31, 55, 66]
Accompanying symptoms [55, 66]	**2**	**2** [55, 66]
Sleep disturbance [56, 76]	**2**	**2** [56, 76]
Sensory processing difficulties [37]	**1**	**1** [37]
Fatigue [76]	**1**	**1** [76]
**Treatment variables**		
Including medication [8, 51, 63, 77, 79]	**5**	**2** [8, 79]
Without medication [25, 29, 39, 41, 44, 69, 70]	**7**	**4** [29, 41, 69, 70]

### Patient characteristics

Relationships between QoL and patient characteristics, namely gender and age, were examined in seven studies in total (Table 3). Three studies found a significant difference in QoL between males and females, in which females scored worse on QoL [35, 51], while one study did not report which gender scored better on QoL [73]. In three of six studies that examined age, significant differences in all QoL subscales were reported, all reporting a better QoL in younger aged patients [53, 61, 73].

### Psychological traits

A total of ten determinants were examined that concern psychological traits (Table 3).

Significant correlations were reported between QoL and depressive symptoms, negative life events, stress, anger, goal frustration, cognitive and health mindset (Table 3). Negative affectivity correlated with physical functioning [49]. Several coping strategies were also examined in relation with QoL: self-blame, acceptance, rumination and catastrophizing were all found to be significantly related with QoL. The coping strategy ‘focus on planning’ was significantly correlated with frequency of headaches (i.e., patients suffering monthly headaches), although no significant correlation was found in patients with weekly headaches [51].

### Headache variables

A total of four headache variables were examined in nineteen studies (Table 3). Ten studies examined type of headache and their relationship with QoL. Most studies focused on migraine and tension-type headache. Results varied strongly between studies (Table 2).

Six studies explored the relations of headache frequency with QoL. Massey et al. reported a poorer QoL in case of increased frequency [51]. Langeveld et al. reported moderate to strong correlations between frequency and QoL regarding functional status, satisfaction with life and satisfaction with health [49]. Powers et al. reported a weak relationship between QoL and headache frequency [61].

Severity or intensity of headaches were examined in seven studies (Table 3). Two studies did not find a relationship between severity and QoL [51, 61], while three studies reported that poorer QoL was associated with headache severity [29, 65, 78]. Nodari et al. found that intensity of the headache attacks was related with subscale scores that assess anxiety and headache impact [56]. Another study found increased headache severity was associated with lower scores on the physical scale of the PedsQL [22].

### Comorbidity and functional impairment

A total of seven determinants that concern comorbidity and functional impairment were identified in twelve studies (Table 3). Disability was examined most often (*n* = 6 studies). Disability was often measured by the Pediatric Migraine Disability Assessment (PedMIDAS). Five studies reported correlations between disability and QoL, indicating disability to be an important determinant for QoL in pediatric patients [23, 29, 37, 54, 55].

Relationships between comorbidities and QoL were presented in three studies [55, 58, 71]. Petersen et. al examined differences between patients suffering headaches with and without other pain conditions in children with weekly and recurrent headache. In both groups, comparison showed a moderate difference in QoL-score [58]. Öztop et al. and Slater et al. both included headache patients with and without psychiatric disorders. Öztop did not find a significant differences in QoL scores, while the study of Slater et al. presented significantly worse QoL in headache patients with a lifetime psychiatric disorder in comparison with patients with headaches that did not have psychiatric diagnoses [55, 71]. Other determinants that were identified included pain measurements, accompanying symptoms in general, sleep disturbance, sensory processing difficulties and fatigue (Table 3).

### Treatment variables

Twelve studies examined determinants of treatment type for headaches (Table 3). Determinants were categorized into pharmacological and non- pharmacological interventions. Five studies examined the relationship with pharmacological interventions and QoL, in which two reported significant improvement of QoL after intervention [55, 71].

Determinants regarding non-pharmacological interventions included seven different interventions. Significant effects regarding improvement of QoL were found for biofeedback and (cognitive behavioral) pain management [29, 69, 70]. Concerning mindfulness, a significant improvement of QoL was measured in parent-reported outcomes. However, this intervention did not result in an improved QoL from the patient perspective [41].

## Discussion

This systematic review identified and assessed PROMs currently used to measure QoL in pediatric patients suffering headaches. No studies were found using a PROM instrument with a good methodological quality, that was specifically developed for measuring QoL in pediatric patients with headaches.

Second, QoL concepts that were important to pediatric patients with headaches were sorted.

Moreover, to identify variables that have been researched previously to explain variability in the QoL of children with headaches, determinants and their relation with QoL were reported. The identified determinants can be used to guide future research concerning the QoL in children with headaches.

The completeness and significance of each sub-construct cannot be measured based on the review. However, the outcomes can be used as a conceptual model, that can function as a fundament for the development of a new PROM instrument.

A preliminary conceptual model was developed that will be used to inform the development of an interview guide and preliminary coding framework for qualitative interviews.

The resultant conceptual model that was devised, is based on findings from this study. Most of the research today has relied on the outcomes of generic QoL questionnaires, primarily the PedsQL. Given the reliance on generic tools, it is possible that important concepts that are specific to pediatric patients with headaches might not have been identified. Further qualitative research is needed to determine the importance of the identified QoL concepts in the PROMs used to date, and whether there are any missing concepts.

The availability of a pediatric headache-specific PROM to assess QoL would facilitate the standardization of outcome measurement across studies and in clinical care to enable the systematic assessment of QoL and benchmarking of outcomes [83]. An important factor for the latter, is to use a clear definition of the patient group(s) included. Most studies included in this review tend to use the IHS classification or the ICHD-II criteria. With the new ICD III criteria available, its use in research-purposes would assure similar definitions and classification of patients between centres and research-groups. Although the ICD III can be very useful for implementation in the daily clinic as well, its criteria are rather strict, which might cause challenges for usage in the daily clinic.

This review provides the first important step in recognizing a range of determinants that form a complex interaction with determinants and the QoL of children suffering headaches.

The majority of the studies included in this review reported relationships between determinants and QoL for an extensive range of factors. However, many determinants were only reported in one or two studies. Especially determinants concerning psychological traits and daily functioning are studied less frequently. Seen that, even in the limited number of studies, their relation with QoL was often significant, these findings may be of importance for future research in this field.

### Limitations

Concerning the identified determinants, we found that the reported values of the relationship between determinants and QoL were often insignificant. As studies were often based on a small sample size, the lack of significant findings might be due to the impaired power to detect clinically important relationships that might exist as a result of the small sample sizes.

It could also be due to the tools themselves, as use of generic tools not specifically designed for pediatric patients with headaches could be measuring the wrong concepts, or asking about concepts in a way that does not resonate with the patient sample. Furthermore, we only reported determinants that are directly related to QoL of children suffering headaches. Indirect relationships with QoL have not been described clearly so far. No studies have reported about the (complex) interrelationships between determinants, such as physical and psychological health, and emphasize an important topic for future research.

Most studies that were reviewed considered pediatric patients suffering migraine and TTH. Importantly, the study of Slater et al. reported psychiatric comorbidities in 29.6% of the included patients with headaches [71]. However, seventeen studies included in this review explicitly excluded patients with psychiatric comorbidities. Since there seems to be less focus on patients with psychiatric disorders suffering headaches, future research should include patients with psychiatric diagnoses, in order to explore their problems and needs. A future QoL measurement instrument should therefore be applicable for usage in this patient group as well.

We found that most studies included patient samples from high-income countries. No studies were conducted in low-income countries, and only two studies examined patients from lower-middle income countries. Consequently, our conceptual model is based on outcomes measured in patient samples from high-income countries. The few studies available on the epidemiology of children suffering headaches in low-income countries report a high incidence [84, 85]. It is unclear if children living in lower-income countries face even more challenges, seen the multidimensionality of poverty and its reported effect on QoL [86, 87]. Future qualitative interviews with children suffering from headaches should include children from lower-income countries too, to determine if it is possible to develop a cross-cultural QoL PROM that could be used internationally.

## Conclusion

Currently there is no content-specific and psychometrically sound PROM available to assess QoL in pediatric patients suffering headaches. Concepts from the included studies in this review were identified and used to form a preliminary conceptual model. The model could be used to inform future qualitative interviews with children suffering from headaches and the development of a comprehensive new QoL PROM. Patients deriving from countries other than high income countries, as well as patients suffering headaches associated with psychiatric disorders were underrepresented in the studies included in this review. Therefore, further steps towards development of a pediatric headache PROM should also include input from patients that are diagnosed with psychiatric disorders, and patients deriving from countries with other income classifications.

### Supplementary Information

**Additional file 1.**

## Data Availability

All data generated or analyzed during this study are included in this published article [and its supplementary information files]. If wished, python codes for the analyses and creation of the figures can be obtained as well.
