# Supplementary material for: Quality of life in children suffering from headaches: a systematic literature review
Source: J Headache Pain. 2023 Sep 18;24(1):127. doi: 10.1186/s10194-023-01595-0 (PMC10506203; doi:10.1186/s10194-023-01595-0)
Supplement: Supplementary file 1 — Additional file 1. [file 10194_2023_1595_MOESM1_ESM.docx]

# **Appendix 1 Search strategy**

## **embase.com**

(‘headache and facial pain’/exp OR migraine/de OR ‘benign paroxysmal vertigo of childhood’/de OR ‘benign paroxysmal torticollis’/de OR ‘intracranial hypertension’/exp OR ‘brain pseudotumor’/de OR (headache* OR migrain* OR cephalgi* OR cephalalgi* OR caephalgi* OR caephalalgi* OR sunct OR suna OR hemicranias OR (trigeminal* NEAR/3 (neuralgi* OR neuropath*)) OR prosopalgi* OR prosoponeuralgi* OR aura OR (cyclic NEAR/3 vomit*) OR (benign* NEAR/3 paroxysmal* NEAR/3 (vertigo* OR torticol*)) OR (idiopath* NEAR/3 (intracranial* OR intra-cranial*) NEAR/3 hypertensi*) OR ((brain OR cerebr*) NEAR/3 (pseudotumo* OR pseudo-tumo*))):ab,ti) AND ((‘validation process’/de OR ‘validation study’/de OR validity/de OR ‘content validity’/de OR ‘quality of life assessment’/exp OR (validat* OR validit* OR ((QoL OR hrql OR QoL OR wellbeing OR well-being) NEAR/6 (assessment* OR measur* OR Index OR Indeces OR improve*)) OR (quality NEAR/3 life NEAR/6 (assessment* OR measur* OR Index OR Indeces))):ab,ti) OR ((‘questionnaire’/exp OR ‘clinical assessment tool’/de OR ‘assessment of humans’/exp OR (questionnair* OR tool OR tools OR scale OR scales OR (scor* NEAR/3 (system))):ab,ti) AND (‘quality of life’/exp OR ‘wellbeing’/de OR ‘psychological well-being’/de OR ‘patient-reported outcome’/de OR ((quality NEAR/3 life) OR QoL OR hrql OR QoL OR wellbeing OR well-being OR patient*-report*-outcome*):ab,ti))) AND (child/exp OR adolescent/exp OR adolescence/exp OR ‘child behavior’/de OR ‘child parent relation’/de OR pediatrics/exp OR childhood/exp OR ‘child nutrition’/de OR ‘infant nutrition’/exp OR ‘child welfare’/de OR ‘child abuse’/de OR ‘child advocacy’/de OR ‘child development’/de OR ‘child growth’/de OR ‘child health’/de OR ‘child health care’/exp OR ‘child care’/exp OR ‘childhood disease’/exp OR ‘child death’/de OR ‘child psychiatry’/de OR ‘child psychology’/de OR ‘pediatric ward’/de OR ‘pediatric hospital’/de OR ‘pediatric anesthesia’/de OR ‘pediatric intensive care unit’/de OR ‘neonatal intensive care unit’/de OR (adolescen* OR preadolescen* OR infan* OR newborn* OR (new NEXT/1 born*) OR baby OR babies OR neonat* OR child* OR kid OR kids OR toddler* OR teen* OR boy* OR girl* OR minors OR underag* OR (under NEXT/1 (age* OR aging)) OR juvenil* OR youth* OR kindergar* OR puber* OR pubescen* OR prepubescen* OR prepubert* OR pediatric* OR paediatric* OR school* OR preschool* OR highschool* OR suckling* OR PICU OR NICU OR PICUs OR NICUs):ab,ti) NOT ([Conference Abstract]/lim AND [1800-2016]/py)

## **Medline Ovid**

(exp Headache/ OR exp Migraine Disorders/ OR Intracranial Hypertension/ OR Pseudotumor Cerebri/ OR (headache* OR migrain* OR cephalgi* OR cephalalgi* OR caephalgi* OR caephalalgi* OR sunct OR suna OR hemicranias OR (trigeminal* ADJ3 (neuralgi* OR neuropath*)) OR prosopalgi* OR prosoponeuralgi* OR aura OR (cyclic ADJ3 vomit*) OR (benign* ADJ3 paroxysmal* ADJ3 (vertigo* OR torticol*)) OR (idiopath* ADJ3 (intracranial* OR intra-cranial*) ADJ3 hypertensi*) OR ((brain OR cerebr*) ADJ3 (pseudotumo* OR pseudo-tumo*))).ab,ti.) AND ((validation process/ OR validation study/ OR validity/ OR content validity/ OR quality of life assessment/ OR (validat* OR validit* OR ((QoL OR hrql OR QoL OR wellbeing OR well-being) ADJ6 (assessment* OR measur* OR Index OR Indeces OR improve*)) OR (quality ADJ3 life ADJ6 (assessment* OR measur* OR Index OR Indeces))).ab,ti.) OR ((questionnaire/ OR clinical assessment tool/ OR assessment of humans/ OR (questionnair* OR tool OR tools OR scale OR scales OR (scor* ADJ3 (system))).ab,ti.) AND (quality of life/ OR wellbeing/ OR psychological well-being/ OR patient-reported outcome/ OR ((quality ADJ3 life) OR QoL OR hrql OR QoL OR wellbeing OR well-being OR patient*-report*-outcome*).ab,ti.))) AND (child/ OR adolescent/ OR adolescence/ OR child behavior/ OR child parent relation/ OR pediatrics/ OR childhood/ OR child nutrition/ OR infant nutrition/ OR child welfare/ OR child abuse/ OR child advocacy/ OR child development/ OR child growth/ OR child health/ OR child health care/ OR child care/ OR childhood disease/ OR child death/ OR child psychiatry/ OR child psychology/ OR pediatric ward/ OR pediatric hospital/ OR pediatric anesthesia/ OR pediatric intensive care unit/ OR neonatal intensive care unit/ OR (adolescen* OR preadolescen* OR infan* OR newborn* OR (new ADJ born*) OR baby OR babies OR neonat* OR child* OR kid OR kids OR toddler* OR teen* OR boy* OR girl* OR minors OR underag* OR (under ADJ (age* OR aging)) OR juvenil* OR youth* OR kindergar* OR puber* OR pubescen* OR prepubescen* OR prepubert* OR pediatric* OR paediatric* OR school* OR preschool* OR highschool* OR suckling* OR PICU OR NICU OR PICUs OR NICUs).ab,ti.) NOT (news OR congres* OR abstract* OR book* OR chapter* OR dissertation abstract*).pt.

## **PsycINFO Ovid**

(exp Headache/ OR (headache* OR migrain* OR cephalgi* OR cephalalgi* OR caephalgi* OR caephalalgi* OR sunct OR suna OR hemicranias OR (trigeminal* ADJ3 (neuralgi* OR neuropath*)) OR prosopalgi* OR prosoponeuralgi* OR aura OR (cyclic ADJ3 vomit*) OR (benign* ADJ3 paroxysmal* ADJ3 (vertigo* OR torticol*)) OR (idiopath* ADJ3 (intracranial* OR intra-cranial*) ADJ3 hypertensi*) OR ((brain OR cerebr*) ADJ3 (pseudotumo* OR pseudo-tumo*))).ab,ti.) AND ((Test Validity / OR validation study / OR (validat* OR validit* OR ((QoL OR hrql OR QoL OR wellbeing OR well-being) ADJ6 (assessment* OR measur* OR Index OR Indeces OR improve*)) OR (quality ADJ3 life ADJ6 (assessment* OR measur* OR Index OR Indeces))).ab,ti.) OR ((Questionnaires / OR Rating Scales / OR (questionnair* OR tool OR tools OR scale OR scales OR (scor* ADJ3 (system))).ab,ti.) AND (“quality of life”/ OR Well Being / OR ((quality ADJ3 life) OR QoL OR hrql OR QoL OR wellbeing OR well-being OR patient*-report*-outcome*).ab,ti.))) AND (100.ag. OR 200.ag. OR (adolescen* OR preadolescen* OR infan* OR newborn* OR (new ADJ born*) OR baby OR babies OR neonat* OR child* OR kid OR kids OR toddler* OR teen* OR boy* OR girl* OR minors OR underag* OR (under ADJ (age* OR aging)) OR juvenil* OR youth* OR kindergar* OR puber* OR pubescen* OR prepubescen* OR prepubert* OR pediatric* OR paediatric* OR school* OR preschool* OR highschool* OR suckling* OR PICU OR NICU OR PICUs OR NICUs).ab,ti.) NOT (news OR congres* OR abstract* OR book* OR chapter* OR dissertation abstract*).pt.

## **CINAHL EBSCOhost**

(MH Headache + OR TI(headache* OR migrain* OR cephalgi* OR cephalalgi* OR caephalgi* OR caephalalgi* OR sunct OR suna OR hemicranias OR (trigeminal* N2 (neuralgi* OR neuropath*)) OR prosopalgi* OR prosoponeuralgi* OR aura OR (cyclic N2 vomit*) OR (benign* N2 paroxysmal* N2 (vertigo* OR torticol*)) OR (idiopath* N2 (intracranial* OR intra-cranial*) N2 hypertensi*) OR ((brain OR cerebr*) N2 (pseudotumo* OR pseudo-tumo*))) OR AB(headache* OR migrain* OR cephalgi* OR cephalalgi* OR caephalgi* OR caephalalgi* OR sunct OR suna OR hemicranias OR (trigeminal* N2 (neuralgi* OR neuropath*)) OR prosopalgi* OR prosoponeuralgi* OR aura OR (cyclic N2 vomit*) OR (benign* N2 paroxysmal* N2 (vertigo* OR torticol*)) OR (idiopath* N2 (intracranial* OR intra-cranial*) N2 hypertensi*) OR ((brain OR cerebr*) N2 (pseudotumo* OR pseudo-tumo*)))) AND ((MH Validation Studies OR MH Instrument Validation OR TI(validat* OR validit* OR ((QoL OR hrql OR QoL OR wellbeing OR well-being) N5 (assessment* OR measur* OR Index OR Indeces OR improve*)) OR (quality N2 life N5 (assessment* OR measur* OR Index OR Indeces))) OR AB(validat* OR validit* OR ((QoL OR hrql OR QoL OR wellbeing OR well-being) N5 (assessment* OR measur* OR Index OR Indeces OR improve*)) OR (quality N2 life N5 (assessment* OR measur* OR Index OR Indeces)))) OR ((MH questionnaires OR TI(questionnair* OR tool OR tools OR scale OR scales OR (scor* N2 (system))) OR AB(questionnair* OR tool OR tools OR scale OR scales OR (scor* N2 (system)))) AND (MH quality of life OR MH Psychological Well-Being OR TI((quality N2 life) OR QoL OR hrql OR QoL OR wellbeing OR well-being OR patient*-report*-outcome*))) OR AB((quality N2 life) OR QoL OR hrql OR QoL OR wellbeing OR well-being OR patient*-report*-outcome*)))) AND (MH child+ OR MH adolescence OR TI(adolescen* OR preadolescen* OR infan* OR newborn* OR (new N1 born*) OR baby OR babies OR neonat* OR child* OR kid OR kids OR toddler* OR teen* OR boy* OR girl* OR minors OR underag* OR (under N1 (age* OR aging)) OR juvenil* OR youth* OR kindergar* OR puber* OR pubescen* OR prepubescen* OR prepubert* OR pediatric* OR paediatric* OR school* OR preschool* OR highschool* OR suckling* OR PICU OR NICU OR PICUs OR NICUs) OR AB(adolescen* OR preadolescen* OR infan* OR newborn* OR (new N1 born*) OR baby OR babies OR neonat* OR child* OR kid OR kids OR toddler* OR teen* OR boy* OR girl* OR minors OR underag* OR (under N1 (age* OR aging)) OR juvenil* OR youth* OR kindergar* OR puber* OR pubescen* OR prepubescen* OR prepubert* OR pediatric* OR paediatric* OR school* OR preschool* OR highschool* OR suckling* OR PICU OR NICU OR PICUs OR NICUs)) NOT PT (news OR congres* OR abstract* OR book* OR chapter* OR dissertation abstract*)

## **web of science**

TS=(((headache* OR migrain* OR cephalgi* OR cephalalgi* OR caephalgi* OR caephalalgi* OR sunct OR suna OR hemicranias OR (trigeminal* NEAR/2 (neuralgi* OR neuropath*)) OR prosopalgi* OR prosoponeuralgi* OR aura OR (cyclic NEAR/2 vomit*) OR (benign* NEAR/2 paroxysmal* NEAR/2 (vertigo* OR torticol*)) OR (idiopath* NEAR/2 (intracranial* OR intra-cranial*) NEAR/2 hypertensi*) OR ((brain OR cerebr*) NEAR/2 (pseudotumo* OR pseudo-tumo*)))) AND (((validat* OR validit* OR ((QoL OR hrql OR QoL OR wellbeing OR well-being) NEAR/5 (assessment* OR measur* OR Index OR Indeces OR improve*)) OR (quality NEAR/2 life NEAR/5 (assessment* OR measur* OR Index OR Indeces)))) OR (((questionnair* OR tool OR tools OR scale OR scales OR (scor* NEAR/2 (system)))) AND (((quality NEAR/2 life) OR QoL OR hrql OR QoL OR wellbeing OR well-being OR patient*-report*-outcome*)))) AND ((adolescen* OR preadolescen* OR infan* OR newborn* OR (new NEAR/1 born*) OR baby OR babies OR neonat* OR child* OR kid OR kids OR toddler* OR teen* OR boy* OR girl* OR minors OR underag* OR (under NEAR/1 (age* OR aging)) OR juvenil* OR youth* OR kindergar* OR puber* OR pubescen* OR prepubescen* OR prepubert* OR pediatric* OR paediatric* OR school* OR preschool* OR highschool* OR suckling* OR PICU OR NICU OR PICUs OR NICUs))) AND DT=(article)

## **Cochrane CENTRAL**

((headache* OR migrain* OR cephalgi* OR cephalalgi* OR caephalgi* OR caephalalgi* OR sunct OR suna OR hemicranias OR (trigeminal* NEAR/3 (neuralgi* OR neuropath*)) OR prosopalgi* OR prosoponeuralgi* OR aura OR (cyclic NEAR/3 vomit*) OR (benign* NEAR/3 paroxysmal* NEAR/3 (vertigo* OR torticol*)) OR (idiopath* NEAR/3 (intracranial* OR intra next cranial*) NEAR/3 hypertensi*) OR ((brain OR cerebr*) NEAR/3 (pseudotumo* OR pseudo next tumo*))):ab,ti) AND (((validat* OR validit* OR ((QoL OR hrql OR QoL OR wellbeing OR well next being) NEAR/6 (assessment* OR measur* OR Index OR Indeces OR improve*)) OR (quality NEAR/3 life NEAR/6 (assessment* OR measur* OR Index OR Indeces))):ab,ti) OR (((questionnair* OR tool OR tools OR scale OR scales OR (scor* NEAR/3 (system))):ab,ti) AND (((quality NEAR/3 life) OR QoL OR hrql OR QoL OR wellbeing OR well next being OR patient* next report* next outcome*):ab,ti))) AND ((adolescen* OR preadolescen* OR infan* OR newborn* OR (new NEXT/1 born*) OR baby OR babies OR neonat* OR child* OR kid OR kids OR toddler* OR teen* OR boy* OR girl* OR minors OR underag* OR (under NEXT/1 (age* OR aging)) OR juvenil* OR youth* OR kindergar* OR puber* OR pubescen* OR prepubescen* OR prepubert* OR pediatric* OR paediatric* OR school* OR preschool* OR highschool* OR suckling* OR PICU OR NICU OR PICUs OR NICUs):ab,ti)

## **Google scholar**

headache|migraine validation|validity “quality*life”|wellbeing|”well-being” adolescents|infants|children
